# Costs of medicines and health care: a concern for Australian women across the ages

**DOI:** 10.1186/1472-6963-13-484

**Published:** 2013-11-20

**Authors:** Emily J Walkom, Deborah Loxton, Jane Robertson

**Affiliations:** 1 School of Medicine and Public Health, The University of Newcastle, Newcastle, Australia; 2 Research Centre for Gender, Health and Ageing, The University of Newcastle, Newcastle, Australia

**Keywords:** Medicines, Affordability, Women’s health, Costs, Qualitative

## Abstract

**Background:**

Evidence from Australia and other countries suggests that some individuals struggle to meet the costs of their health care, including medicines, despite the presence of Government subsidies for low-income earners. The aim of our study was to elucidate women’s experiences with the day to day expenses that relate to medicines and their health care.

**Methods:**

The Australian Longitudinal Study on Women’s Health (ALSWH) conducts regular surveys of women in three age cohorts (born 1973–78, 1946–51, and 1921–26). Our data were obtained from free text comments included in surveys 1 to 5 for each cohort. All comments were scanned for mentions of attitudes, beliefs and behaviours around the costs of medicines and health care. Relevant comments were coded by category and themes identified.

**Results:**

Over 150,000 responses were received to the surveys, and 42,305 (27%) of these responses included free-text comments; 379 were relevant to medicines and health care costs (from 319 individuals). Three broad themes were identified: costs of medicines (33% of relevant comments), doctor visits (49%), and complementary medicines (13%). Age-specific issues with medicine costs included contraceptive medicines (1973–78 cohort), hormone replacement therapy (1946–51 cohort) and osteoporosis medications (1921–26 cohort). Concerns about doctor visits mostly related to reduced (or no) access to bulk-billed medical services, where there are no out-of-pocket costs to the patient, and costs of specialist services. Some women in the 1973–78 and 1946–51 cohorts reported ‘too much income’ to qualify for government health benefits, but not enough to pay for visits to the doctor. In some cases, care and medicines were avoided because of the costs. Personal feelings of embarrassment over financial positions and judgments about bulk-billing practices *(‘good ones don’t bulk-bill’*) were barriers to service use, as were travel expenses for rural women.

**Conclusions:**

For some individuals, difficulty in accessing bulk-billing services and increasing out-of-pocket costs in Australia limit affordability of health services, including medications. At greatest risk may be those falling below thresholds for subsidised care such as self-funded retirees and those on low-middle incomes, in addition to those on very low incomes, who may find even small co-payments difficult to manage.

## Background

There is mounting evidence of the struggle for some individuals and families to meet the costs of health care, including medicines. In a Commonwealth Fund survey of seven countries, 37% of US participants and 26% of Australian participants reported either not filling a prescription or skipping a dose, not visiting the doctor, or missing medical tests, treatments, or follow-up because of cost in the previous 12 months (greater than in Germany, New Zealand, Canada, the UK and the Netherlands) [1,2]. Similar behaviours have been reported in other surveys of Australian and US patients [3,4].

In Australia, the publicly funded Pharmaceutical Benefits Scheme (PBS) aims to provide universally affordable access to prescription medicines. All patients contribute to the cost of their PBS medicines via taxation and graded co-payments. There are two categories of patients, general beneficiaries (who paid up to AU$35.40 per prescription item in 2012) and concession or health care card holders (including senior citizens and those in receipt of social security support, who paid reduced contributions of AU$5.80 per prescription item in 2012) [5]. Medical services are available to all Australian citizens under the national insurance scheme, Medicare, which covers all service costs for doctors who ‘bulk-bill’. Bulk-billing doctors do not charge the patient an additional fee, and accept the Medicare rebate as full payment for the consultation [6]. Patients may pay additional out-of-pocket amounts for doctors who do not bulk-bill (i.e. who charge more than the agreed schedule fee) [7].

Even with this universal health insurance coverage through Medicare and Government subsidised prescription costs, out-of-pocket costs for medical care in Australia are increasing [8]. Patient expenditure on all prescription medicines increased from an average of approximately 0.1% of household consumption expenditure in 1971 to 0.43% in 2007 [9]. Increases in patient co-payments for medicines and visits to medical practitioners have increased the financial burden on individuals in recent years [10], especially for visits to specialist practitioners [11]. Expenditure on complementary and alternative medicines (CAM) in Australia is also increasing, and was estimated at over AUD$4 billion in 2005 [12]. The costs of CAM are not subsidised by Government, and use is skewed towards individuals with higher household incomes [13], and with private health insurance [12].

Particularly vulnerable to increases in out-of-pocket medical expenses are those with chronic illness [2,8,14], and those with comorbidities or who use multiple medications [15]. Heisler *et al.*[16] reported that women were more likely than men to underuse medicines due to costs. Kemp *et al.*[17] reported that medicines underuse due to costs in Australia was significantly higher in younger (18–29 year olds) and mid-aged people (30–64 years), compared to those aged 65 and older; which is understandable given that older Australians are entitled to greater Government subsidies for their medicines. However, other studies have reported that the financial burden for older Australians is still high, despite access to these subsidies [18]. Government concessions on health care costs for those on low incomes do not entirely protect individuals from out-of-pocket burden or financial hardship [10,14]. Jeon *et al. *[15] and Doran *et al. *[3] suggest that there may be a subgroup of patients who are particularly vulnerable to increasing health care costs: those who earn too much to qualify for government subsidies, yet not enough to afford the out-of-pocket costs of their medicines or doctor’s visits.

There are few qualitative studies in the Australian setting that capture the attitudes and experiences of individuals regarding the costs of medicines and health care. One investigation of financial pressures due to illness involved interviews of patients with chronic illness and their carers [15]. High out-of-pocket costs incurred in the treatment of chronic illness were a concern for many participants, including the cost of medicines, consultations with general practitioners and specialists, diagnostic tests, and transportation or parking costs. Participants who were eligible for government subsidised medications expressed gratitude for the scheme (the PBS), stating that they would not otherwise be able to afford their medicines. Another interview study reported that although costs might delay a visit to the doctor until absolutely necessary, the expenses involved with the visit and with any prescribed medicines were not a concern for the majority of the participants [3].

The present study investigates concerns about medicines costs in three age cohorts of women taking part in the Australian Longitudinal Study on Women’s Health (ALSWH). Qualitative comments collected as part of this study provide a rich source of information on topics pertinent to the respondents, and thus offer a patient-centred insight into the concerns and attitudes of women regarding their health and well-being. Byles *et al. *[19] reported on quantitative aspects of the use and costs of medicines and other health care resources for women in the ALSWH, and illustrated their findings with selected comments from survey respondents. We wished to examine these free-text comments systematically and in greater detail with the aim of the study to elucidate women’s experiences with the day to day expenses that relate to medicines and their health care.

## Methods

Data for this study were drawn from the Australian Longitudinal Study on Women’s Health (ALSWH). The ALSWH is an ongoing survey of health and health service use in Australia involving over 40,000 women of three nationally representative age cohorts (named by year of birth: 1973–78 cohort, 1946–51 cohort and 1921–26 cohort). These women were randomly selected from the Medicare Australia database in 1996, and have been followed up by mail surveys occurring on a three yearly staggered cycle by age cohort. Surveys included in this study commenced in 1996 and continued until 2009. The cohorts were intentionally oversampled for those living in rural and remote areas. Detailed methods of the ALSWH are available elsewhere [20]. The ALSWH received ethical approval from the University of Newcastle Ethics Committee (approval number: H-076-0795) and the University of Queensland Medical Research Ethics Committee (approval number: 200400224). While the surveys do contain questions about the costs of visits to the doctor and private health insurance, there are no items that directly address the cost of medications.

Our data were obtained from free text comments returned with the first five surveys for each of the three age cohorts. At the end of each of the ALSWH surveys, participants were asked, “Have we missed anything? If there is *anything* else you would like to tell us, please write on the lines below”. The comments are not prompted by questions, allowing women to comment on any aspect of their health or wellbeing. We scanned all these comments for any mention of attitudes, beliefs and behaviours around the costs of medicines and health care. Following methods used in previous studies [21], an inductive approach was taken for analysis. The comments from each survey were reviewed by two researchers (EW, JR) and code names were assigned to relevant phrases, sentences or passages. Similar codes were grouped by category, and a reflective process was used to identify themes within the categories, with comments reviewed several times to ensure consistency in coding. Selected (de-identified) comments are presented to illustrate the views of the women and are labelled by cohort (Y, M, O for 1973–78 [younger], 1946–51 [mid-aged] and 1921–26 [older] cohorts respectively, and by survey number 1–5) with minor typographical and spelling errors corrected for ease of reading.

## Results

### Comments

Over 150,000 responses were received to the five surveys of each of the three age cohorts, and 42,305 (27%) of these responses included free-text comments. The 1973–78, 1946–51 and 1921–26 cohorts provided 26%, 31% and 43% of these comments respectively (Table 1). Topics covered by the comments were wide-ranging, but women in all age cohorts commented on their personal health issues, consultations with medical practitioners, preference (or otherwise) for female doctors, attitudes towards taking medications in general, adverse effects of some medicines, and difficulties in accessing health care services, particularly in regional and rural areas. Age-specific issues with medications were also reported, for example: contraceptive medicines for the 1973–78 cohort, hormone replacement therapy for the 1946–51 cohort and osteoporosis medications for the 1921–26 cohort. Some women commented that the ALSWH surveys did not directly address the issue of health care costs.

**Table 1:** Number of comments from surveys of each age cohort

	**1973-1978 responses**		**1946-1951 responses**		**1921-1926 responses**	
**Survey**	**Comments/Total**	**%**	**Comments/Total**	**%**	**Comments/Total**	**%**
1	2423/14762	16.4	2447/14072	17.4	2978/12804	23.3
2	1948/9688	20.1	2058/12338	16.7	4695/10434	45.0
3	2267/9081	25.0	2672/11226	23.8	3955/8647	45.7
4	2056/9145	22.5	2966/10905	27.2	3573/7158	49.9
5	2415/8200	29.5	2915/10638	27.4	2937/5560	52.8
TOTAL	11109/50876	21.8	13058/59179	22.1	18138/44603	40.7

Of the 42,305 comments, 379 were identified as covering topics that related to the research questions and were included in the current analysis, 28% from the 1973–78 cohort, 50% from the 1946–51 cohort and 22% from the 1921–26 cohort. The included comments were from 319 individuals, with some women making relevant comments in more than one survey.

Most of the survey comments considered by the researchers to contain remarks relevant to costs of medicines and related health care could be divided into three broad themes: doctor visits (49% of relevant comments), medicines (33%), and complementary medicines (13%, Table 2). Women from the 1946–51 cohort were more likely to comment on health care costs than those in the 1973–78 and 1921–26 cohorts.

**Table 2:** Number of comments relevant to costs of medicines and health care

**Cohort**	**Total**	**Medicines N (%)**	**Doctor visits N (%)**	**Complementary medicines N (%)**	**Other N (%)**
1973-78	106	22 (20.8%)	72 (67.9%)	10 (9.4%)	2 (1.9%)
1946-51	188	53 (28.2%)	85 (45.2%)	30 (16%)	20 (10.6%)
1921-26	85	49 (57.6%)	27 (31.8%)	8 (9.4%)	1 (1.2%)
**Total**	379	124 (32.7%)	184 (48.5%)	48 (12.7%)	23 (6.1%)

### Costs of doctor visits

Women from all age cohorts raised concerns with the reduction or lack of bulk-billing (no out-of-pocket cost to patient) medical services in their area. “*No doctors in [large rural town] bulk-bill, the ones who do are full*” (Y5); *“Over the last 12 months two local practices have stopped bulk-billing … It is increasingly harder to find a doctor that bulk-bills and I object to paying a $17 gap for just a prescription and almost no consultation”* (M3). Conversely, other participants chose to deliberately avoid bulk-billing doctors, reporting concerns with the perceived quality of care from bulk-billing practices, particularly large medical centres. “*If you want a reasonable GP you have to pay extra as the good ones don’t bulk-bill. The ones that do bulk-bill treat you like cattle…”* (Y2).

Difficulties in affording visits to non-bulk-billing doctors were mentioned by all age groups: respondents from the 1973–78 cohort (particularly students) mentioned lack of income and government support as barriers to access: “*No Austudy [student welfare payment] (or any other form of government support), therefore no money available to see doctors and such unless it is a dire emergency”* (Y1). The 1921–26 cohort referred to their pensioner status as a reason for being unable to afford doctor visits: “*Small country town medical clinics do not give bulk-billing to aged pensioners and insist on cash payment on the day of visit.… Many pensioners would not seek medical help when needed if at the time no cash was available*” (O1). “*This last 6 months money has become a little tight as we are both attending the Dr quite regularly and with the clinic we attend not bulk-billing we are required to pay $3.50 per visit… the extra cost per week in the last 2 or 3 months has been approximately $15-$20; this sure makes a hole in the pension…*” (O1). “*Charges for re-writing scripts are expensive, at least $3 to $5, this is not claimable”* (O3). The added burden of claiming a partial refund from Medicare for each non-bulk-billed consultation was mentioned by some of the 1921–26 cohort: “*… I don’t like to complain, please forgive me, but since bulk-billing and increase in lots of chemist prices, it is more difficult. We now have to make extra trip to Medicare to collect some of the fee back”* (O3).

In the 1946–51 cohort, women mentioned embarrassment over their financial position, and the inability to claim back money spent on visits to the doctor. “*Reasons for not asking professional help for many minor ailments are cost and time of transport to services and also cost of medication etc. - if possible I keep away from doctors, dentists etc. because of cost and subsequent embarrassment because of my poor financial position”* (M2). “*I intend to put up with minor complaints and not visit the doctor mainly because I have to pay for my visit and am unable to get back the gap even though I have private health cover*.” (M1). “*I feel that a lot of women ignore their health due to financial circumstances. When you are a low income earner and have to save to visit a doctor, it certainly makes life difficult. If all doctors bulk-billed it would make it easier or at least let you claim on Medicare and then pay the difference instead of demanding payment in advance. For myself I would like to go and have a full check-up but it is financially impossible*” (M1).

Some women in the 1973–78 and 1946–51 cohorts reported earning ‘too much income’ to qualify for government health benefits, but not enough to pay for visits to the doctor: “*…we earn too much apparently to have a heath care card, but we don’t earn a lot to afford to pay to see a doctor. … The government has no idea what kind of pressures this puts on mothers*” (Y3). “*… Although I earn just over 11 dollars per hour I am not eligible for a health care card so I’m precluded from pap smears and other medical services”* (M2). “*Because of our assets I am not entitled to any form of assistance but each week the cost of my medications, doctor’s visits, tests and X-ray do not help our cash situation*” (M4).

Comments from the 1946–51 and 1921–26 cohorts mentioned the specific difficulties faced by self-funded retirees. *“My husband and I are self-funded retirees. We have saved and forgone holidays over many years to avoid the need for a pension, and lower interest rates now affect our income. Many people in our situation avoid seeking medical attention even when it would be advisable, because of the big difference in the “excess of the prescribed fee” that the doctors charge - and this also for specialists, radiographers, etc. that may follow on. This means that free Medicare is a myth*” (O1).

Women of all ages in regional and rural areas reported not only increased difficulty in accessing necessary health care services in their area, but also increased costs associated with travel and overnight stays, when health care services were located far away from their homes. *“… I saw a specialist and had [tests] at a base hospital. In the days of bulk-billing (alas, no more) this service was free, but the last two episodes have cost $40 apiece. This together with cost of petrol to travel 156 km two days running, makes this an expensive treatment*” (O3).

Some respondents had difficulties affording the cumulative costs of attending a doctor to obtain a prescription then buying the medicine: *“… We have great problems being able to afford 30–70 dollar doctor’s fees even if you can claim it back. We have to rush around to Medicare to get it back so we can get through the week. … We then have to weigh up if we can afford any prescribed medicines”* (Y1). “*… Some weeks I don’t have a spare $40 to see a doctor and another $20 or so to buy medicine. This has caused me some stress and concern over my health in the last 12 months*” (Y3).

### Costs of medicines

Women of all ages mentioned the costs of prescription medicines in their comments. The specific topics covered differed for each of the age cohorts surveyed.

Many of the comments from the 1973–78 cohort relating to the costs of medicines concerned the cost of the contraceptive pill, whether used for contraceptive purposes or for management of other conditions: “*…the reason I am not taking the pill right now is I don’t want to pay to go to the GP or for the pills*” (Y3). “*Currently not on the pill as the one I take (for contraception and other health reasons) is too expensive*” (Y3). Some younger respondents mentioned other particular medicines: “*Annoyed that medicines that would help me (if they work they put the prices up!) are so expensive - Arthritis medicine, Flu shots and even Cold Sore Cream!*” (Y2). One respondent from the 1973–78 cohort wrote of her reliance on government-subsidised prescriptions: “*I rely heavily on those government scripts. They are great otherwise I would be at the chemist every 2 weeks. I get 4 packets at once. It’s all authorised through my doctor. It would also cost me a fortune if I wasn’t on a health care card. I don’t know how those who aren’t cope*” (Y3).

Women in the 1946–51 cohort mentioned the on-going cost of medications for chronic illnesses: “*… Frustratingly most of the problems I encounter require medication for life. No condition is really in and of itself, life threatening but all are a reminder of an ageing body. Current medications cost approx. $190 or more per month”* (M3). “*…Prescriptions (e.g. eye drops, which I have to use twice daily for the rest of my life) should be less expensive if a woman has to use something on a long term basis.*” (M1). “*Having experienced epilepsy for 35 years - … My drugs cost over $60 per month. As my husband is in work we have no health care card*” (M1). *“I feel it is important you know that being single on a low income with no other support, buying my home … I cannot afford to pay for all my scripts for asthma and allergy … So if I neglect my health it is purely because of financial difficulty”* (M2).

In the later surveys of the 1946–51 cohort, the issue of non-subsidised osteoporosis medication was raised: “*Osteoporosis – on-going medication and screening very expensive if not entitled to health care card and finance Private Health Insurance - Essential HRT and some medication not on PBS e.g. [Medicine name] very expensive if not entitled to Health* [Care] *Card”* (M4). “*.... My bone is worse I need [Medicine name] but I can’t afford to pay $68 a month for 4 tablets.*” (M5). Other non-subsidised medicines were also mentioned: “*… I have started treatment with [skin cancer cream]. … is not on the “pharmaceutical list”. I am a d.s* [disability support] *pensioner total income $175 per week the 3 tubes of cream cost $132, so I couldn’t buy food*” (M2). “*… I also use cheaper medication (for migraine) because the cost of the better medication is prohibitive ($60 for 2 tablets) and there is a limit to the pharmaceutical benefits I can claim in a 12 month period”* (M1).

Hormone replacement therapy was a hot topic for women in the 1946–51 cohort, but most comments concerned side effects, or the choice between prescription hormone replacement therapy and alternative treatment methods. A few women did raise issues around the costs of treatment: “*… I think the price of HRT is high as it is not an optional medicine it is a necessity*” (M2).

For women in the 1921–26 cohort, the high cost of medicines in general was an issue for both self-funded retirees and for women receiving a pension. Some women were unhappy that their income precluded them from receiving a Health Care Concession Card, which reduces the cost of subsidised prescription medicines. “*I feel dissatisfied with attitude of Health Department in dealing with self-funded retirees. I earn just too much to get a Commonwealth Health Card through thrifty living and wise investment, but have high on-going medication expenses*” (O1).

Even with concessions on medication prices, it was still too much to pay for some women. “*I would like to see all pensioners get free medicine, jabs etc. free instead the safety net as once you have $140.00 you get it free. But until you make the $140.00 it is a struggle … I need more than two scripts a fortnight so I have to pay the extra which gives you a choice of you have medication or food”* (O1).

Some medications are not eligible for any subsidy or government concessions: *“… having to pay extra for medication because generic brands do not help to keep severe [condition] … under control. The [medication name] … the most controllable medication I have used. My problem is that they used to cost me $2.70 and now have jumped to $12.35. Plus other medications go up in price quite often. I get a pension rise and the government take it back and more this way*” (O1). “*The doctor put me on a capsule called [medication name]. These capsules are $100 for 100. I take 2 capsules per day which means they only last fifty days. The government does not help with the cost of these which makes it very expensive.”* (O2). *“I have to be in hospital when I get [type of infection] which requires intravenous antibiotics. Some of them are very expensive which I take home orally, one is $200 the other is $90 = $290 per month, that is more than one week’s pension, without help from my daughter, I would not be able to have these drugs”* (O3).

As with the 1946–51 cohort, the older women also mentioned the cost of non-subsidised osteoporosis medications. “*I have to pay full price for tab [Medicine name]. I haven’t had a break or bone fracture, only pain from past injuries. I wonder why this medication can’t be available to patients over a certain age, before they do have a break or fracture to bones filling up the hospitals and nursing homes. It could be cheaper for the government in the long run*” (O4) “*… I went on to a new treatment called [name]. The specialist thinks well of this 18 month treatment and it seems to be working, but it is expensive (total cost over 18 months $15,000) and is not on the pharmaceutical benefits, so if you can’t pay, too bad”* (O5). “*I was prescribed [Medicine name] quite a long while ago but due to the scare about it having bad side effects such as gum infection I was taken off it by my doctor. There is no medication prescribed under the PBS scheme for osteoporosis even though it is life threatening”* (O5).

### Costs of complementary medicines

Women in the 1946–51 cohort were more likely than women in the 1921–26 or 1973–78 cohorts to mention issues with the cost of complementary medicines in their comments. Few younger women had comments in this area. Respondents expressed a desire for the costs of complementary medicines to be government subsidised: “*…It is frustrating that medical funds do not refund as much for “alternative” treatments which in some cases are most effective”* (M1). “*… The cost of natural supplements such as herbs, vitamins minerals, should be covered by health funds or government assistance. It costs me about $150 per month for natural supplements, on which ability to earn a living depends.*” (M2). “*… Naturopath understanding and trying to help - but it’s difficult for one on limited income, as I can’t use my Health Care Card at Naturopath.”* (M3). “*Unfortunately vitamins etc. are becoming almost as expensive as medication. They should be subsidised to help older people afford them…”* (O2). *“Is there any way herbal medicines can be put on the pharmaceutical list?”* (O2).

## Discussion

While the majority of Australians appear to have reasonable access to healthcare, our data show that difficulties in accessing bulk-billing (no cost to patient) services and increasing out-of-pocket costs have had a detrimental impact on some women’s ability to afford health services, including medications (see also Young and Dobson 2003 [6]). Based on comments from our survey, groups that may be at particular risk, are those who just miss out on thresholds for subsidised care (health care cards) such as self-funded retirees and those on middle incomes, in addition to those on very low incomes, such as students, who meet threshold requirements but find even minimal co-payments to be out of reach. Further research is required to explore this further. Personal feelings of embarrassment over their financial positions as well as judgments about the value of bulk-billing practices were also barriers to service use, as were travel expenses for women living in non-urban areas.

Many of the aspects raised by the women were common to all age cohorts, while others were relevant to each particular stage of life. Women in the 1946–51 cohort may be more likely to earn too much money to qualify for financial assistance compared with younger women, who may still be students, or older women, who are more likely to receive the age pension and associated concessions. Women from the 1946–51 cohort also mentioned the burden of medicines for long-term treatment or chronic illness, supporting findings from previous research [14,15]. Women from the 1946–51 and 1921–26 cohorts both mentioned the costs of osteoporosis treatment, and the cumulative costs of multiple medications.

Almost half of the relevant comments made by participants concerned the costs of visits to medical practitioners. Concerns were raised by women from all age cohorts about the lack of access to fully-subsidised consultations with medical practitioners (bulk-billing). Substantial out-of-pocket expenses are incurred when doctors charge more than the scheduled fee (which is Government funded) – especially for those with multiple comorbidities. Comments from the first three surveys of each age cohort were from a time when bulk-billing rates were at a historic low; media reports at the time refer to rapid growth in out-of-pocket fees for patients [22,23]. Government reforms to the Medicare system in 2003 introduced incentives to medical practitioners to increase bulk billing rates, particularly in rural and remote areas, as well as in metropolitan areas with lower rates of bulk-billing [24]. Bulk-billing rates have been increasing since these reforms to a high of around 81% Australia-wide in March 2013 [25]. Bulk billing rates are now higher in remote areas than in major cities. Nevertheless, there were still comments in the surveys conducted post-2003 reforms that indicate that lack of access to bulk-billing doctors remains a concern for some women. Furthermore, some women voiced a lack of confidence in the quality of care received at bulk-billing “mega-clinics,” or drop-in medical centres (“*The ones that do bulk-bill treat you like cattle…*”).

There are safety-nets which reduce or remove patient co-payments once a certain level of out-of-pocket spending has been reached, but many households struggle before they meet these minimum levels of spending [14]. As shown in previous research [14,18], women eligible for a concession or pension card were not shielded from the burden of high out-of-pocket healthcare costs. This is a concern if women are facing difficulties with the costs of health professional visits, as this is only the first step in accessing prescription medicines.

The Australian health care system provides mechanisms that go some way to protect the most vulnerable against financial barriers to access – those on low incomes, with social security support, and retired persons more likely to have multiple comorbidities. But others continue to struggle – the “working poor,” who despite paid employment [26], find resources are strained trying to meet out-of-pocket expenses, such as medical and prescription costs [27]. As demonstrated in a number of settings, the response for some is to avoid seeking care, choosing not to get prescriptions dispensed, or not taking medicines as directed [1-3], with poorer health outcomes as a result [16]. Policies that increase patient contributions and out-of-pocket expenses may be consistent with ‘user pays’ principles that are designed to encourage quality use of medicines and help Governments meet their budgetary targets; however there can be unintended consequences - along with compromised health care and health outcomes, there is family stress and anxiety as families make uncomfortable choices about prioritising medical needs and choosing between food, doctor visits and prescription medicines.

The use of complementary and alternative medicines is growing [12]. There were many comments made by participants in the ALSWH from all age cohorts regarding perceptions of benefits and preference for CAM over conventional prescription medicines (although these were not examined in detail in this study), and some women questioned why access to these medicines was not subsidised by taxpayers. The cost of CAM has been noted as a barrier to access in other studies of mid-aged women during menopause [28]. A challenge for the manufacturers of these medicines is to assemble the clinical data to support claims of the cost-effectiveness of CAM. Demonstration of cost-effectiveness is a prerequisite for listing on the Australian PBS [29].

There are some limitations with our data. Only a very small minority of women in the ALSWH identified the costs of medicines and health care as a concern, and caution is warranted in interpreting these comments as being representative of the wider population. Nevertheless, there were sufficient responses across all cohorts to indicate cost is an important consideration for some women and across the ages. These comments were spontaneous; the women were not directly asked to comment on health care costs, and yet it was important enough for them to write down and share their experiences with ALSWH investigators. Health care costs were a problem despite the existence of universal health insurance (Medicare) and Government subsidises (concession cards for low income earners and safety nets) designed to mitigate the costs of medical consultations and prescription medicines for Australian families.

Our sample consisted only of women. However, women tend to make most of the health care decisions for their family [30], and tend to be more frequent users of health care services in general, particularly women of childbearing age [31]. Women may have more frequent contact with health care providers due to a greater utilisation of preventive health care [32]. Although the ALSWH respondents have generally been shown to be broadly representative of the Australian population, there is some response bias towards women with tertiary education and under-representation of some groups of immigrant women [33]. What this means for our data is that cost concerns may be under-reported, if the sample is skewed towards women who are likely to have higher incomes. There has been some attrition in later surveys of each age cohort; however the number of comments has not reduced proportionally (Table 1). In addition, withdrawal from the on-going surveys often relates to being burdened with illness and other health and family issues; those women may be more likely to be struggling with health care costs. However, responses to the open-ended invitation to comment on any aspect of health may be biased toward the negative [34].

Some of the specific issues raised by participants in the surveys, conducted between 1996 and 2009, may no longer be concerns due to policy changes. For example, members of the 1921–26 cohort mentioned the strict restrictions on access to subsidised osteoporosis medications; these restrictions have since been relaxed and more women are now able to access the medicines through the PBS at a greatly reduced personal cost. However, there are on-going concerns about access to other new and often expensive medicines in the Australian community. Studies on the impact of increased out-of-pocket (co-payment) medicine costs suggest that the increased costs negatively impact on use of common and important chronic use medicines such as statins [35].

## Conclusions

Australia’s healthcare system is designed to reduce financial barriers to access, but there are still groups who incur proportionally large out-of-pocket costs, particularly those with multiple comorbidities and chronic conditions. Concession or pensioner status only goes some way to ameliorate this burden. Our examination of comments provided spontaneously from women of all age groups show that these issues of affordability continue to be reported over time and affect women of all ages.

## Competing interests

The authors declare the following competing interests: Deborah Loxton holds a current Australian Research Council (ARC) linkage grant with Family Planning New South Wales and Bayer (Pharmaceutical company), investigating contraceptive use. Jane Robertson and Emily Walkom declare that they have no competing interests.

## Authors’ contributions

EW and JR conceived of the study, participated in its design and coordination, analysed the data and helped to draft the manuscript. DL participated in the design of the study and helped to draft the manuscript. All authors read and approved the final manuscript.

## Pre-publication history

The pre-publication history for this paper can be accessed here:

http://www.biomedcentral.com/1472-6963/13/484/prepub
